# Adjuvant administration of hypertonic saline in lumbar epidural intervention may be associated with successful response in patients with probable neuropathic radicular pain Screened by Douleur Neuropathique 4

**DOI:** 10.7150/ijms.59695

**Published:** 2021-05-17

**Authors:** Yongsoo Lee, Sunmin Kim, Jin-Woo Shin, Jeong-Gil Leem, Seong-Soo Choi

**Affiliations:** 1 Department of Anesthesiology and Pain Medicine, Uijeongbu Eulji Medical Center, Eulji University School of Medicine, Uijeongbu-Si, Republic of Korea.; 2 Department of Anesthesiology and Pain Medicine, Asan Medical Center, University of Ulsan College of Medicine, Seoul, Republic of Korea.

**Keywords:** neuropathic pain, chronic pain, radicular pain, lumbar, Douleur Neuropathique 4, hypertonic saline

## Abstract

**Background:** Chronic lumbar radicular pain often accompanies neuropathic pain. The treatment may follow a screening for probable neuropathic pain rather than the definitive diagnosis, which is often difficult in daily practice. However, interventional management may have limited effects on symptoms in patients with neuropathic radicular pain refractory to conservative treatments. The purpose of this study is to evaluate the factors associated with successful responses after lumbar epidural intervention in patients with chronic lumbar neuropathic radicular pain determined by Douleur Neuropathique 4 (DN4).

**Methods:** We retrospectively reviewed 221 chronic lumbar radicular pain patients using a DN4 questionnaire prior to the epidural interventional procedure. The patients were divided into two groups according to the DN4 questionnaire: <4-point DN4 and ≥4 DN4. The numerical rating scale (NRS) for pain intensity, changes in physical functional status, and the use of pain medication were obtained before and 1 month after the procedure. Successful responder was defined based on robust combination of outcome parameters. The factors associated with successful response were analyzed using univariate and multivariate regression.

**Results:** We found 170 (76.9%) patients with DN4 <4 and 51 (23.1%) with a score ≥4. Among the total 221 patients, 129 (58.4%) were successful responders and 92 (41.6%) were non-responders regardless of DN4 score. We observed a significantly lower proportion of successful responders among patients with a DN4 score ≥4 (22, 43.1%) than patients with a score <4 (107, 62.9%) (P=0.012). After adjusting in multivariate regression analysis, the DN4 score was independently associated with response after lumbar epidural intervention (odds ratio [OR]=0.838; 95% confidence interval [CI]=0.718-0.978; P=0.025). In subgroup logistic regression analysis according to the DN4 score, adjuvant administration of hypertonic saline during epidural interventions in patients with a DN4 score ≥4 (OR=3.71; CI=1.142-12.457; P=0.029) was associated with the success of the lumbar epidural procedure at 1 month.

**Conclusion:** The adjuvant use of hypertonic saline in lumbar epidural interventions may be effective at least 1 month after the intervention in patients with probable neuropathic lumbar radicular pain ≥4 using the DN4.

## Introduction

Chronic lower back pain (LBP) or lower leg radicular pain is one of the leading causes of poor quality of life 1, 2. Conservative management is the first-line treatment for chronic lumbar radicular pain; this includes exercise, oral therapy, physiotherapy, and epidural injections 3-5. Neuropathic components often ensue in patients with chronic lumbar radicular pain, and the interventional treatment for neuropathic radicular pain is limited to symptom management 6, 7. If conservative treatments fail to relieve symptoms, surgical intervention could be considered. However, according to a study comparing patients who underwent surgery and non-surgical treatment, surgery is not significantly more advantageous than non-surgical treatment 8-10. Therefore, it is important to seek to improve the outcome of lumbar interventions that will help manage patients with neuropathic lumbar radicular pain.

According to the International Association for the Study of Pain, neuropathic pain is pain arising as a direct consequence of a lesion or disease affecting the somatosensory system 11. However, its definitive diagnosis is often difficult. In daily practice, the diagnosis of the type of pain precedes that of the somatosensory lesion 12. Therefore, specifically developed screening questionnaires are useful in identifying probable neuropathic pain 13. Among several neuropathic screening tools, the Douleur Neuropathique 4 (DN4) is a useful questionnaire to screen neuropathic pain. Thus, in the present study, we examined the factors related to successful response to lumbar epidural intervention in patients with chronic neuropathic radicular pain determined by the DN4.

## Materials and Methods

This retrospective study was conducted at the pain clinic of our institute. The necessity for informed consent was waived as only recorded data were reviewed. We reviewed the electronic medical records of patients for all necessary data that were itemized and recorded at their visits to the pain clinic. This study protocol was approved by our institutional review board (approval number 2020-1538), and the study was conducted in accordance with the Declaration of Helsinki.

### Patients

We reviewed the records of patients who first visited our pain clinic from January to December 2016. All aspects of patient privacy and confidentiality were preserved. Patients were included in the present study if they met the following criteria: 1) were at least 20 years of age; 2) had chronic radicular pain and/or lower back pain for more than 3 months; 3) symptoms were not relieved or had not subsided with exercise, medical treatment, or physiotherapy; 4) they used a DN4 questionnaire at the first visit; and 5) they underwent epidural interventional procedure after the first visit. We excluded the patients who had any of the following conditions: 1) age <20 years; 2) acute pain for less than 3 months; 3) signs of progressive neurological deficits or motor weakness; 4) allergies to steroids or contrast dyes; 5) uncontrollable or unstable opioid use; 6) coagulopathy; 7) systemic or injection site infection; 8) unstable medical or psychiatric condition; 9) malignancy; 10) lost to follow-up; and 11) incomplete medical records. Patients were divided according to the DN4 questionnaire: <4-point DN4 group and ≥4-point DN4 group. Patients with a DN4 score ≥4 were considered as having a probable neuropathic pain 14.

### Procedure

The lumbar epidural procedures included in this study were lumbar epidural block, caudal epidural block, transforaminal epidural block, percutaneous epidural adhesiolysis with or without a balloon catheter, and pulsed radiofrequency treatment.

### Parameters for Outcome Assessments

At the first visit to our institution, we determined the DN4 score of all patients with lumbar radicular pain prior to outpatient treatment. Also, patients were taught to use an 11-point NRS (0 = no pain and 10 = worst possible pain) to assess the intensity of their lumbar radicular pain with or without LBP. The patients were routinely followed up in the outpatient department of our pain clinic 1 month after the lumbar epidural procedures. On follow-up, the NRS pain score and improvement in physical functional status were obtained.

The improvement of functional status was judged based on the patient's interview at follow-up visit. It was considered as an improvement in functional status when the patient reported that there was an improvement in daily function at the follow-up visit 1 month after the procedure. In addition, data for pain medication use were collected before and 1 month after the procedure. Based on the World Health Organization (WHO) analgesic ladder, we defined the decrease in the use of pain medication as when the step went down in the WHO analgesic ladder after the procedure compared to before the procedure 15. The global perceived effect (GPE) 1 month after the procedure according to a 7-point Likert scale was also measured for evaluation of the patient's satisfaction and overall improvement from baseline after the procedure.

Data, including age, sex, body mass index, duration of pain, neuropathic component, underlying disease, changing of the medicine, and lumbar magnetic resonance image findings, were collected form electronic medical records.

### Definition of Treatment Response

According to previous studies, we defined the responder group with some modifications 16-18: (1) a decrease in pain intensity of more than 4-point or 50% on the NRS; or (2) a decrease in pain intensity of more than 2-point or 30% on the NRS with simultaneous improvement of functional status from baseline and decreased medication.

### Statistical Analysis

Continuous demographic data from <4-point DN4 and ≥4 DN4 groups were compared using the Student's t-test. Categorical demographic data were compared using the chi-square test. By using univariate and multivariate regression, the factors associated with successful response 1 month after the epidural intervention were analyzed. The most relevant factors associated with successful responses were included in the univariate logistic regression analysis. The inclusion of variables in the final multivariate logistic regression analysis to evaluate independent factors associated with treatment responses was based on the biological plausibility, clinical importance, and statistical considerations (p <0.2). The quality of fit of the model was assessed with the Hosmer-Lemeshow test. A two-tailed p-value <0.05 was considered statistically significant. The data were analyzed using SPSS software version 21.0 (SPSS, Inc., Chicago, IL, USA).

## Results

**Figure 1:**
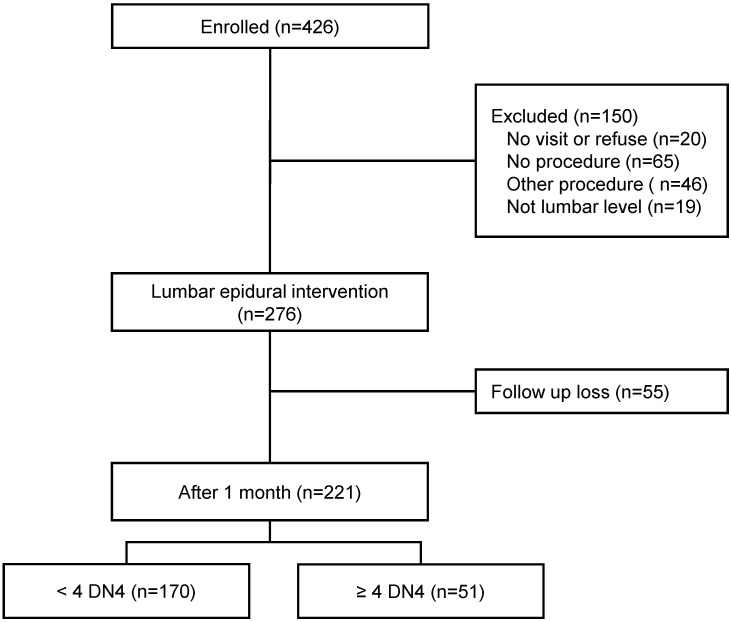
The study flow diagram. DN4: Douleur Neuropathique 4.

From January to December 2016, we enrolled 426 patients who visited our pain clinic with a chief complaint of chronic radicular pain and/or lower back pain for more than 3 months and had a DN4 score (Fig. 1). Initially, 150 patients were excluded before the lumbar epidural intervention. Twenty patients did not visit after the first visit or refused the epidural intervention, 65 were treated with a drug instead of receiving an epidural procedure, 46 patients benefitted from another procedure (medial branch block or transcutaneous electrical nerve stimulation), and 19 patients underwent the epidural procedure at a different level (thoracic or cervical level). Patients who had successful lumbar epidural interventions numbered 276. We excluded 55 patients who did not come to our pain clinic after 1 month. Thus, 221 patients were included and followed up 1 month after the lumbar epidural intervention.

We found that 170 (76.9%) patients had DN4 <4 and 51 (23.1%) had DN ≥4. The overall baseline demographic characteristics of the 221 patients are shown in Table 1. The median DN4 score in these patients with chronic lumbar radicular pain was 2.0 (1.0-3.0). The common symptoms differed between groups (P=0.019). Radicular leg pain was observed more in patients with a DN4 score ≥4 (Table 1). Diagnosis in these patients differed between groups according to the DN4 (P=0.017). The pain intensity on NRS did not differ between groups (P=0.133). There were no significant differences in other variables. Table 2 shows the characteristics of lumbar epidural interventions. There were no significant differences in the DN4 score between groups.

According to the definition described above, there were 129 (58.4%) responders and 92 (41.6%) non-responders among all patients regardless of DN4 score. However, their demographic and interventional characteristics did not differ (Supplementary Table 1 and 2). Of the patients with a DN4 score <4, 107 (62.9%) had a successful response 1 month after lumbar epidural intervention. However, we found significantly fewer successful responders among patients with a DN4 score ≥4 (22, 43.1%, P=0.012, Table 3). The observed numbers of patients in the two groups who satisfied the individual parameters for a successful response at each follow-up visit are listed in Table 3 and Supplementary Table 3. No significant differences were detected between the 2 groups. In addition, GPE scores of <4 DN4 group and ≥4 DN4 group were 4.0 (4.0-6.0) and 4.0 (4.0-6.0) without significant difference (P=0.671).

Table 4 shows the univariate and multivariate regression analyses of factors associated with successful response 1 month after epidural intervention in all patients. Variables were selected considering biological plausibility, clinical importance, and statistical considerations. In univariate logistic regression analysis, we identified the following factors as having a meaningful statistical p-value below 0.2 (P <0.2): diagnosis (P=0.105), pain intensity (P=0.133), and DN4 (P=0.089) (Supplementary Table 1). After adjusting in multivariate regression analysis, only the DN4 score was independently associated with response 1 month after the lumbar epidural intervention (odds ratio [OR]=0.838; 95% confidence interval [CI]=0.718-0.978; P=0.025).

In addition, we performed subgroup analysis by DN4 scores to investigate factors associated with successful response 1 month after epidural intervention (Table 5). On subgroup logistic regression analysis according to the DN4 score, variables were selected considering their biological plausibility, clinical importance, and statistical considerations (P < 0.2), as seen in Tables 1 and 2. We found that adjuvant administration of hypertonic saline epidurally in patients with a DN4 score of ≥4 was associated with successful response of the lumbar epidural procedure at 1 month (OR=3.771; CI=1.142-12.457; P=0.029).

## Discussion

This study including patients with chronic lumbar radicular pain demonstrated that patients with a DN4 score ≥4 had significantly fewer successful responses 1 month after lumbar epidural interventions than those with a DN4 score <4. However, we found that adjuvant administration of hypertonic saline in lumbar epidural interventions might be associated with the successful response 1 month after the procedures, although these patients had a DN4 score ≥4.

Considering the challenge in diagnosing neuropathic pain in daily clinical practice, screening tools for neuropathic pain are important to manage patients with possible neuropathic pain. The DN4 questionnaire is an important screening tool for neuropathic pain, and it has a high sensitivity and specificity in distinguishing neuropathic pain from chronic non-neuropathic pain 19-22. The DN4 consists of 10 items, 7 items related to the pain quality (sensory and pain descriptors) and collected through an interview with the patient and 3 items based on the clinical examination 19. At a DN4 score ≥4, the DN4 is highly sensitive (83%) and specific (90%) to diagnose chronic neuropathic pain 19. Compared with other screening questionnaires, the DN4 sensory test component is considered better as it can be self-administered, is convenient, and leads to decreased cost and effort 23. Therefore, we believe that the DN4 can be a suitable screening tool in daily practice.

After screening and identifying probable neuropathic pain, treatment is usually started before the definitive diagnosis of chronic neuropathic radicular pain 7, 24, 25. The treatment of these patients can be complex, and patients usually experience only a partial relief of pain, or appeal to intolerable side effects. Less than half of the patients with neuropathic pain benefit from pharmacologic agents 26, 27. For these reasons, interventional treatments, ranging from a simple nerve block to epidural neuroplasty, are often preferred 10. Nikolanjen et al. explained that early aggressive control of pain may reduce chronic neuropathic pain risk and decrease pain severity 28. Epidural steroid injection is a commonly used intervention to treat chronic spinal pain in patients with radiculopathy. However, there is no evidence to support that multiple injections will generate long-term pain relief in patients with radicular neuropathic pain 29. A previous systematic review gave a weak recommendation for the use of epidural steroid injection in patients with radiculopathy, based on fair evidence of moderate benefit for short-term outcomes 30. It also concluded that there is insufficient evidence to recommend a specific treatment strategy (approach technique, use of steroid during procedure) 30. Interestingly, we found that adjuvant administration of hypertonic saline in lumbar epidural intervention was a factor associated with successful response after the lumbar epidural procedure at 1 month in patients with probable neuropathic pain. These results suggest that adjuvant administration of hypertonic saline during lumbar epidural interventions can improve the success rate in patients with probable lumbar neuropathic radicular pain.

Hypertonic saline (hyperosmolar sodium chloride) is sometimes used as an adjuvant for epidural interventional procedures 31-35. Adding 5-10% hypertonic sodium chloride during lumbar epidural interventions is effective and provides significant pain relief for at least 1 month. Previous studies found that when hypertonic saline was injected as an adjuvant in patients with refractory chronic radiculopathy, the effect of epidural intervention was maintained for at least 3 months 36, 37. Taken together with the present results, it is thought that even in patients with possible neuropathic pain, if adjuvant hypertonic saline is administrated during the lumbar epidural intervention, pain may be reduced for at least 1 month. Although the exact mechanism of pain relief by hypertonic saline has not been fully understood, it can be explained by the neuromodulation effects of chloride solutions and the effect of hyperosmolar solutions on nerve conduction 38, 39. King et al. 38 reported that chloride ions play an important role in establishing a persistent C-fiber blockade, which can be observed when dorsal roots are exposed to hypertonic sodium chloride. In addition, hyperosmolar solutions affect the signal propagation and the compound action potential amplitude of A-fibers in rat dorsal root ganglion 39; thus, it is assumed that the hyperosmolarity of the administered sodium chloride solution may contribute to changes in pain conductivity. Further studies are required to elucidate the mechanism of pain processing by hypertonic saline.

There were some limitations in this study. First, this study was retrospective, with restricted data collection. Therefore, a randomized controlled trial should be conducted to evaluate the effect of adjuvant hypertonic saline on lumbar epidural interventions in patients with chronic neuropathic radicular pain. Second, the follow-up period was short. Although patients were evaluated at 1 month in the present study, it would be important to consider the characteristics of neuropathic pain that did not likely respond to the intervention. Third, we included neuroplasty in the lumbar interventional procedure. When performing neuroplasty, hypertonic saline administration is key to the procedure. Therefore, combining neuroplasty with a complex mixture of solutions and mechanical factors may be a bias to the effects of adjuvant hypertonic saline. Finally, the result may vary according to the definition of successful response. Because the criteria of the successful response included the report from the patient interview, subjective factors could influence the success response might lead to different results.

## Conclusion

In conclusion, the adjuvant use of hypertonic saline in the lumbar epidural intervention may be effective at least 1 month after the intervention in patients with probable neuropathic lumbar radicular pain graded DN4 ≥4.

## Supplementary Material

Supplementary tables.

## Figures and Tables

**Table 1:** Baseline characteristics according to the DN4 questionnaire

Variables	<4 DN4 (N=170)	≥4 DN4 (N=51)	*P* value
Age (years)	67.0 (58.0-75.0)	64.0 (51.0-73.0)	0.087
Sex (male/female)	107 (62.9%)/63 (37.1%)	29 (56.9%)/22 (43.1%)	0.434
BMI (kg/m^2^)	24.5 ± 3.2	25.1 ± 3.7	0.319
Diabetes	30 (17.6%)	13 (25.5%)	0.215
Hypertension	69 (40.6%)	25 (49.0%)	0.285
Spondylolisthesis	26 (15.3%)	5 (9.8%)	0.369
**Symptom**			0.019
Back pain	54 (31.8%)	6 (11.8%)	
Radicular leg pain	38 (22.4%)	15 (29.4%)	
Both	78 (45.9%)	30 (58.8%)	
**Diagnosis**			0.017
Disc herniation	34 (20.0%)	5 (9.8%)	
Spinal stenosis	89 (52.4%)	24 (47.1%)	
Axial cause	15 (8.8%)	5 (9.8%)	
PLSS	29 (17.1%)	11 (21.6%)	
CRPS	1 (0.6%)	4 (7.8%)	
Others	2 (1.2%)	2 (3.9%)	
Pain intensity (NRS)	8.0 (6.0-8.0)	8.0 (6.0-9.0)	0.133
DN4	1.0 (1.0-2.0)	5.0 (4.0-6.0)	<0.001

Data are expressed as mean ± standard deviation, median with interquartile range, or number (%). BMI, body mass index; CRPS, complex regional pain syndrome; DN4, douleur neuropathique 4 questionnaire; NRS, numerical rating scale; PLSS, post lumbar surgery syndrome.

**Table 2:** Characteristics of lumbar epidural interventions according to the DN4 questionnaire

Variables	<4 DN4 (N=170)	≥4 DN4 (N=51)	*P* value
Target levels			0.626
1 level	162 (95.3%)	50 (98.0%)	
2 levels	6 (3.5%)	1 (2.0%)	
3 levels	2 (1.2%)	0 (0.0%)	
**Epidural interventions**			0.235
Simple epidural block	128 (75.3%)	34 (66.7%)	
Balloon neuroplasty	35 (20.6%)	12 (23.5%)	
Neuroplasty without balloon	7 (4.1%)	5 (9.8%)	
Use of hypertonic saline	47 (27.6%)	19 (37.3%)	0.189

Data are expressed as number (%). DN4, douleur neuropathique 4 questionnaire.

**Table 3:** Observed number of patients who satisfied the individual outcome parameters for a successful response 1 month after lumbar epidural interventions

Variables	<4 DN4 (N=170)	≥4 DN4 (N=51)	*P* value
≥ 50% (or ≥ 4-point) reduction in NRS	68 (40.0%)	17 (33.3%)	0.417
≥ 30% (or ≥ 2-point) reduction in NRS	105 (61.8%)	29 (56.9%)	0.530
Functional improvement	103 (60.6%)	22 (43.1%)	0.036
Decreased medication	30 (17.6%)	7 (13.7%)	0.669
Successful responder*	107 (62.9%)	22 (43.1%)	0.012

*Successful response was defined as: 1) ≥50% (or ≥4-point) reduction from the baseline numerical rating scale of pain intensity; or 2) ≥30% (or ≥2-point) reduction from the baseline numerical rating scale of pain intensity with simultaneous improvement of the functional status from baseline and decreased medication use. DN4, douleur neuropathique 4 questionnaire; NRS, numerical rating scale.

**Table 4:** Logistic regression analysis of factors associated with successful response 1 month after epidural intervention

Variables	Univariable			Multivariable		
	OR	95% CI	*P*	OR	95% CI	*P*
**Diagnosis**						
Disc herniation	1 (Ref)			1 (Ref)		
Stenosis	0.947	0.444-2.019	0.887	0.998	0.464-2.144	0.995
Axial	0.840	0.277-2.545	0.758	0.893	0.290-2.752	0.843
PLSS	0.414	0.167-1.024	0.056	0.441	0.176-1.103	0.080
CRPS	0.140	0.014-1.378	0.092	0.208	0.020-2.119	0.185
Others	1.680	0.159-17.719	0.666	2.219	0.203-24.262	0.514
Pain intensity	0.871	0.737-1.028	0.102			
DN4	0.825	0.711-0.958	0.012	0.838	0.718-0.978	0.025

CI, confidence interval; CRPS, complex regional pain syndrome; DN4, douleur neuropathique 4 questionnaire; OR, odds ratio; PLSS, post lumbar surgery syndrome.

**Table 5:** Subgroup logistic regression analysis of factors associated with successful response 1 month after epidural intervention according to the DN4 questionnaire

Variables	<4 DN4			≥4 DN4		
	OR	95% CI	*P*	OR	95% CI	*P*
Age	0.999	0.976-1.023	0.965	0.973	0.937-1.009	0.143
**Symptom**						
Back pain	1 (Ref)			1 (Ref)		
Leg pain	2.240	0.911-5.508	0.079	2.286	0.316-16.512	0.413
Both	1.352	0.667-2.739	0.403	1.333	0.210-8.462	0.760
**Diagnosis**						
Disc herniation	1 (Ref)			1 (Ref)		
Stenosis	0.895	0.386-2.074	0.795	1.773	0.249-12.599	0.567
Axial	0.717	0.204-2.525	0.605	2.250	0.179-28.254	0.530
PLSS	0.589	0.211-1.642	0.311	0.150	0.010-2.289	0.172
CRPS	0.000	0.000-NC	0.999	0.500	0.028-8.952	0.638
Others	0.478	0.027-8.380	0.478	NC	0.000-NC	0.999
Pain intensity	0.930	0.767-1.128	0.460	0.779	0.549-1.107	0.163
Hypertonic saline	1.054	0.524-2.119	0.882	3.771	1.142-12.457	0.029

CI, confidence interval; CRPS, complex regional pain syndrome; DN4, douleur neuropathique 4 questionnaire; NC, not calculated; OR, odds ratio; PLSS, post lumbar surgery syndrome.

## Abbreviations

DN4: Douleur Neuropathique 4
NRS: numerical rating scale
GPE: global perceived effect
LBP: lower back pain
WHO: World Health Organization
