# Supplementary material for: Adjuvant administration of hypertonic saline in lumbar epidural intervention may be associated with successful response in patients with probable neuropathic radicular pain Screened by Douleur Neuropathique 4
Source: Int J Med Sci. 2021 May 17;18(12):2736–42. doi: 10.7150/ijms.59695 (PMC8176191; doi:10.7150/ijms.59695)
Supplement: Supplementary file 1 — Supplementary tables. [file ijmsv18p2736s1.pdf]

Supplementary materials

Supplementary Table 1. Baseline characteristics

Variables	Total (N=221)	Non-responder (n=92)	Responder (n=129)	P value
Age (years)	67.0 (57.0–75.0)	68.0 (57.5–75.0)	66.0 (56.0–75.0)	0.592
Sex (male/female)				0.779
Male	85 (38.5%)	34 (37.0%)	51 (39.5%)	
Female	136 (61.5%)	58 (63.0%)	78 (60.5%)	
BMI (kg/m^2)	24.7 ± 3.3	24.8 ± 3.1	24.6 ± 3.4	0.756
Diabetes	43 (19.5%)	21 (22.8%)	22 (17.1%)	0.285
Hypertension	94 (42.5%)	43 (46.7%)	51 (39.5%)	0.286
Spondylolisthesis	31 (14.0%)	15 (16.3%)	16 (12.4%)	0.410
Symptom				0.250
Back pain	60 (27.1%)	28 (30.4%)	32 (24.8%)	
Radicular leg pain	53 (24.0%)	17 (18.5%)	36 (27.9%)	
Both	108 (48.9%)	47 (51.1%)	61 (47.3%)	
Diagnosis				0.105
Disc herniation	39 (17.6%)	14 (15.2%)	25 (19.4%)	
Spinal stenosis	113 (51.1%)	42 (45.7%)	71 (55.0%)	
Axial cause	20 (9.0%)	8 (8.7%)	12 (9.3%)	
PLSS	40 (18.1%)	23 (25.0%)	17 (13.2%)	
CRPS	5 (2.3%)	4 (4.3%)	1 (0.8%)	
Others	4 (1.8%)	1 (1.1%)	3 (2.3%)	
Pain intensity (NRS)	8.0 (6.0–8.0)	8.0 (6.0–8.5)	8.0 (6.0–8.0)	0.133
DN4	2.0 (1.0–3.0)	2.0 (1.0–4.0)	2.0 (1.0–3.0)	0.089
DN4 ≥ 4	51 (23.1%)	29 (31.5%)	22 (17.1)	0.012

Data are expressed as mean ± standard deviation, median with interquartile range, or number (%). BMI, body mass index; CRPS, complex regional pain syndrome; DN4, douleur neuropathique 4 questionnaire; NRS, numerical rating scale; PLSS, post lumbar surgery syndrome.

Supplementary Table 2. Characteristics of lumbar epidural interventions

Variables	Total (N=221)	Non-responder (n=92)	Responder (n=129)	P value
Target levels				0.486
1 level	212 (95.9%)	89 (96.7%)	123 (95.3%)	
2 levels	7 (3.2%)	3 (3.3%)	4 (3.1%)	
3 levels	2 (0.9%)	0 (0.0%)	2 (1.6%)	
Epidural interventions				0.488
Simple epidural block	162 (73.3%)	71 (77.2%)	91 (70.5%)	
Balloon neuroplasty	47 (21.3%)	16 (17.4%)	31 (24.0%)	
Neuroplasty without balloon	12 (5.4%)	5 (5.4%)	7 (5.4%)	
Use of hypertonic saline	66 (29.9%)	24 (26.1%)	42 (32.6%)	0.371

Data are expressed as number (%).

Supplementary Table 3. Observed number of patients who WHO analgesic ladder was decreased 1 month after lumbar epidural interventions

		**<4 DN4 (N=170)**	**≥4 DN4 (N=51)**	**P-value**
Baseline	No	64 (37.6%)	19 (37.3%)	0.848
	WHO step I	81 (47.6%)	22 (43.1%)	
	WHO step II	14 (8.2%)	6 (11.8%)	
	WHO step III	11 (6.5%)	4 (7.8%)	
1 month	No	74 (44%)	20 (39.2%)	0.669
	WHO step I	74 (44%)	21 (41.2%)	
	WHO step II	13 (7.6%)	5 (9.8%)	
	WHO step III	9 (5.3%)	5 (9.8%)	

Data are expressed as number (%).
